# Supplementary figures and images for: Epigenetic clock analysis and increased plasminogen activator inhibitor-1 in high-functioning autism spectrum disorder
Source: PLoS One. 2022 Feb 3;17(2):e0263478. doi: 10.1371/journal.pone.0263478 (PMC8812940; doi:10.1371/journal.pone.0263478)

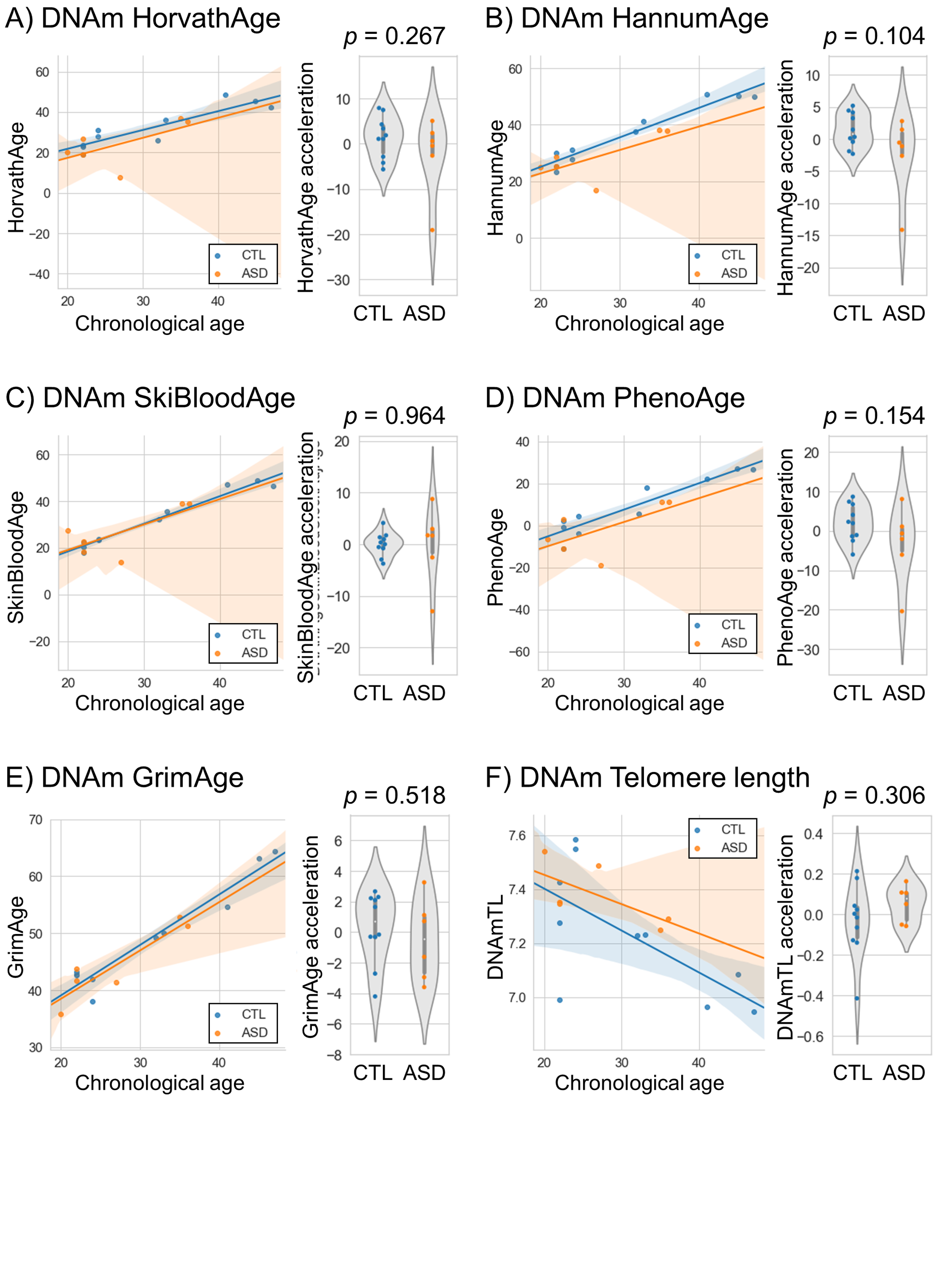

Supplement: S1 Fig — (A) HorvathAge, (B) HannmuAge, (C) SkinBloodAge, (D) PhenoAge, (E) GrimAge, and (F) DNAmTL. Scatter plots show the epigenetic age vs. chronological age. Violin plots with dots show epigenetic age acceleration in the ASD and control groups. Student’s t-tests were performed for comparisons between the groups. ASD, autism spectrum disorder; CTL, control; DNAmTL, DNA methylation-based telomere length. (TIF) [file pone.0263478.s001.tif]

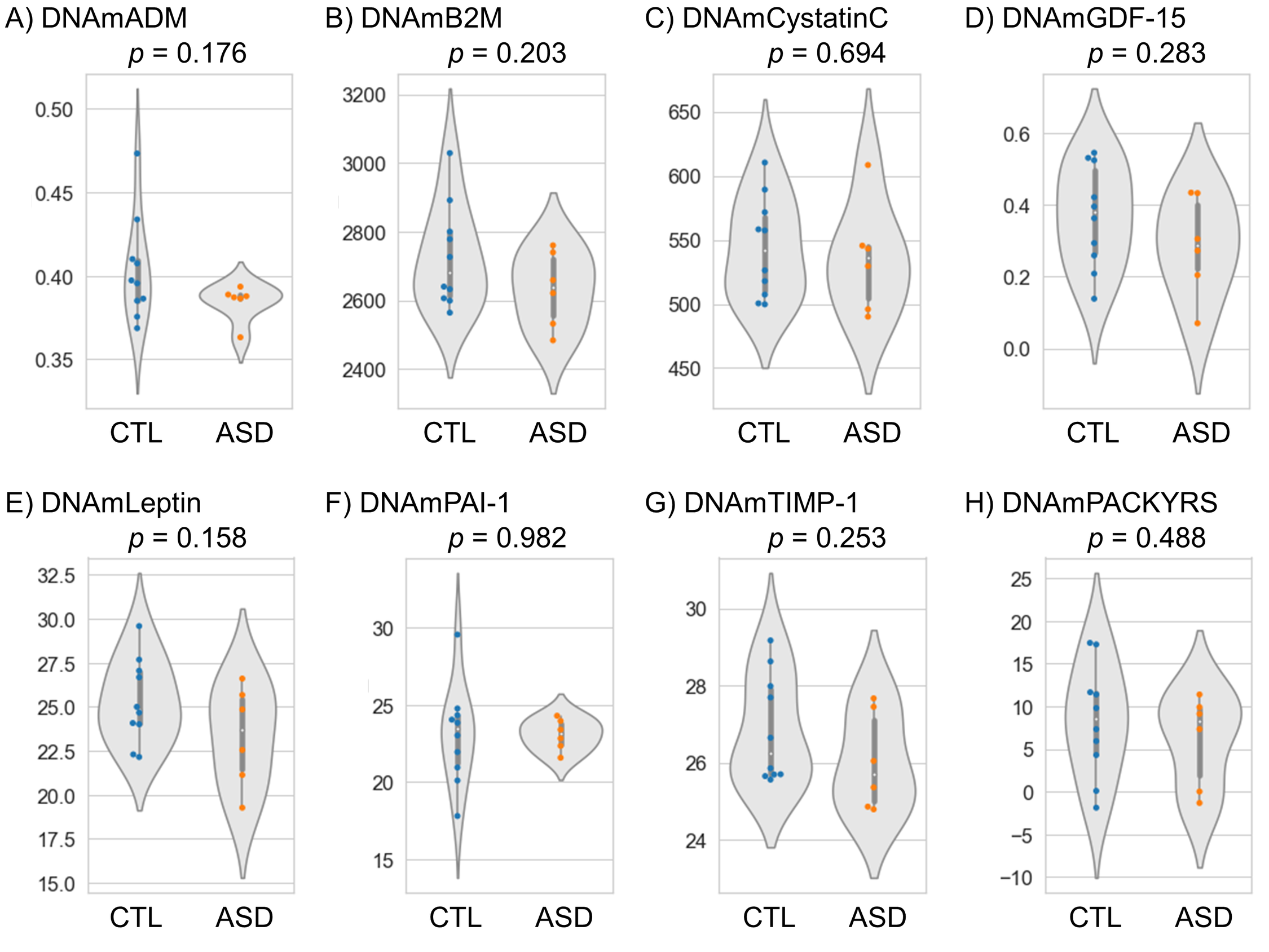

Supplement: S2 Fig — (A) ADM, (B) B2M, (C) Cystatin C, (D) GDF-15, (E) Leptin, (F) PAI-1, (G) TIMP-1, and (H) PACKYRS. Student’s t-tests were performed for comparisons between the groups. ADM, adrenomedullin; ASD, autism spectrum disorder; B2M, beta-2-microglobulin; CTL, control; GDF-15, growth differentiation factor 15; PACKYRS, smoking pack-years; PAI-1, plasminogen activator inhibitor-1; TIMP-1, tissue inhibitor of metalloproteinases-1. (TIF) [file pone.0263478.s002.tif]

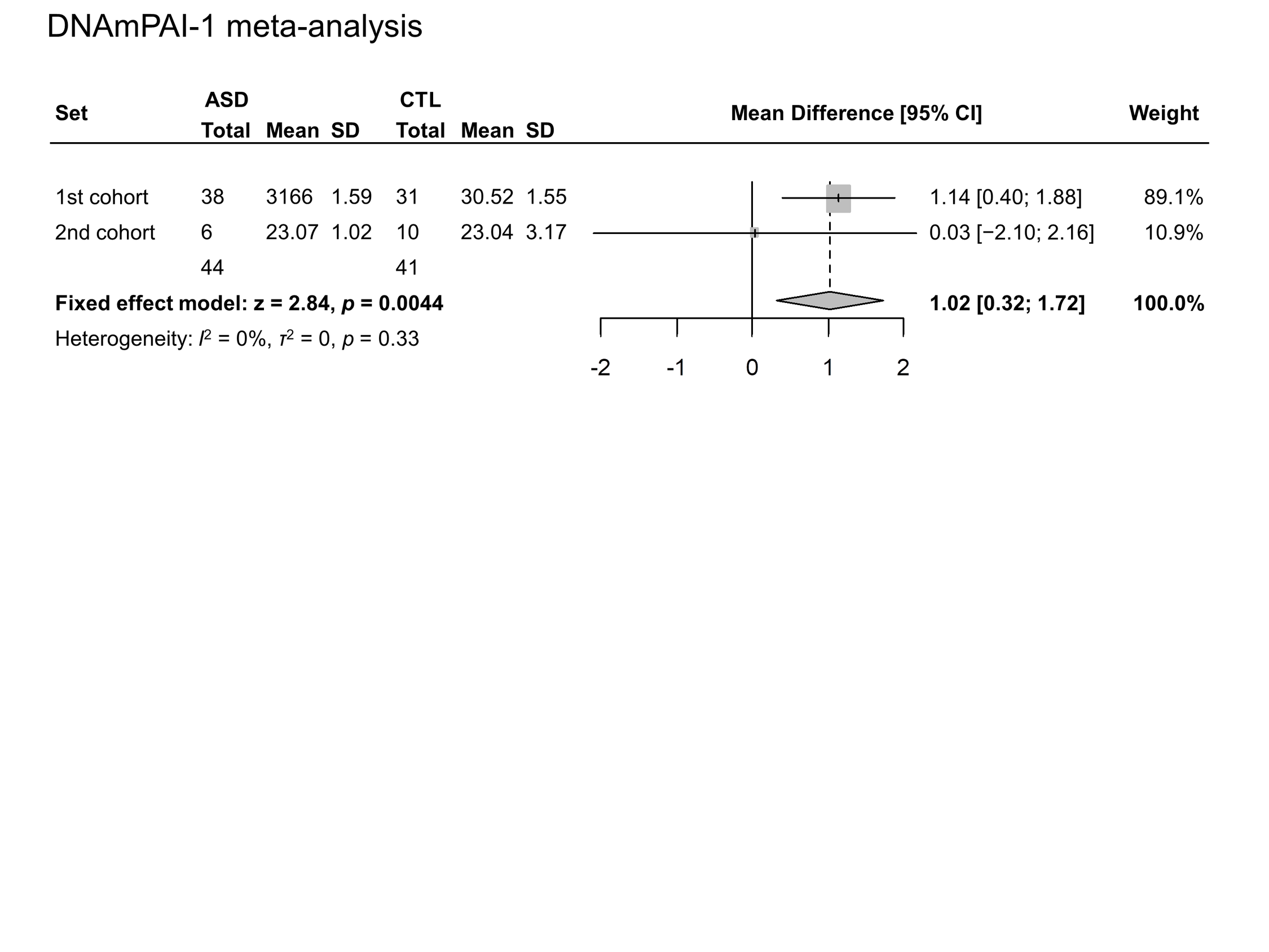

Supplement: S3 Fig — Low heterogeneity was observed between the 1st and 2nd cohorts (Cochran’s Q test p = 0.33). A significant difference was observed between the ASD and control groups using a fixed effect model (p = 0.0044). ASD, autism spectrum disorder; CI, confidence interval; CTL, control; PAI-1, plasminogen activator inhibitor-1; SD, standard deviation. (TIF) [file pone.0263478.s003.tif]

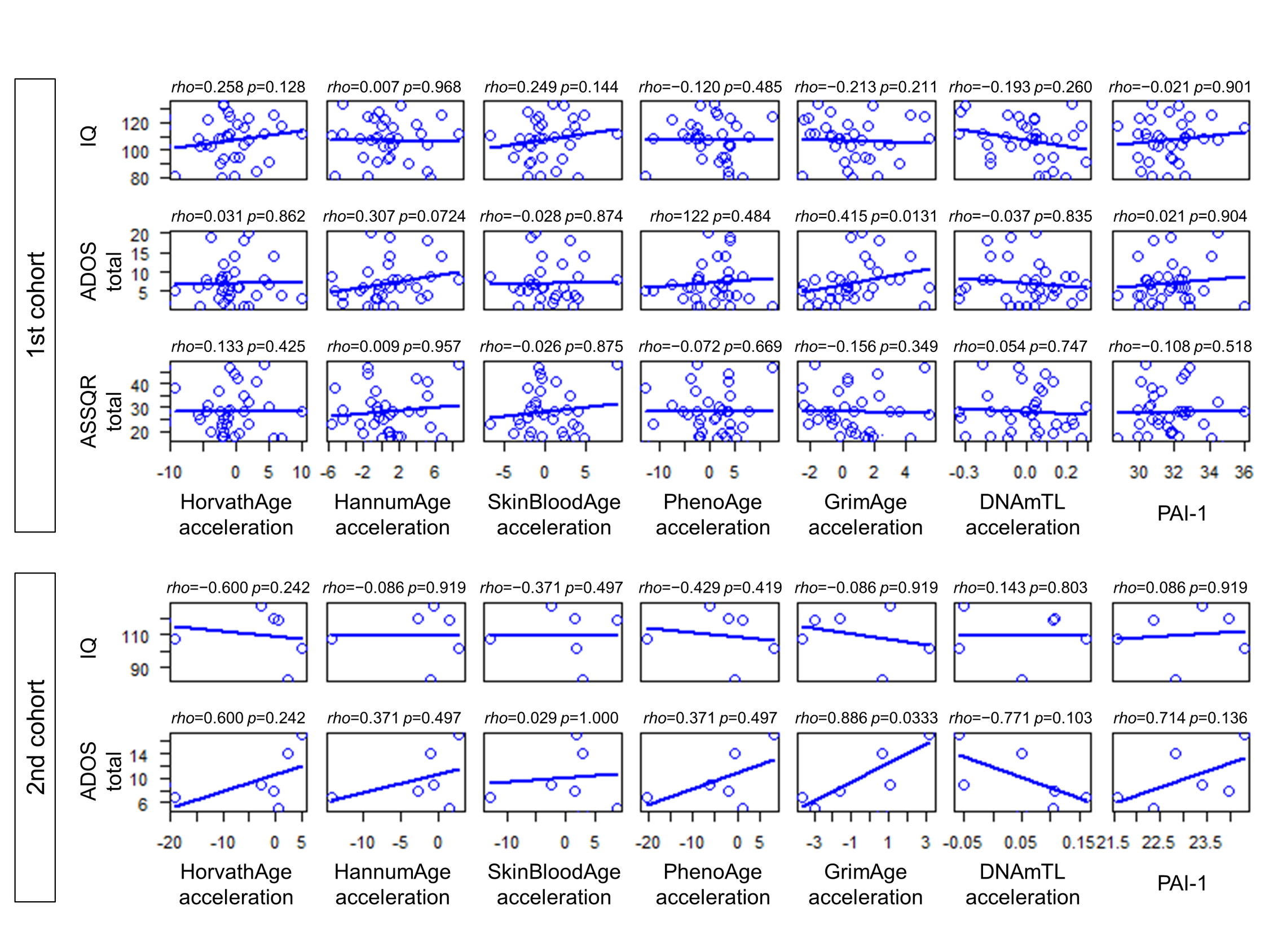

Supplement: S4 Fig — Scatter plots show epigenetic clock acceleration/PAI-1 vs. medical condition. The relationship was analyzed with Spearman’s rank correlation coefficient. ADOS, the Autism Diagnostic Observation Schedule; ASD, autism spectrum disorder; ASSQ-R, the high-functioning Autism Spectrum Screening Questionnaire; CTL, control; DNAmTL, DNA methylation-based telomere length; IQ, intelligence quotient; PAI-1, plasminogen activator inhibitor-1. (TIF) [file pone.0263478.s004.tif]
