# Supplementary material for: Epigenetic clock analysis and increased plasminogen activator inhibitor-1 in high-functioning autism spectrum disorder
Source: PLoS One. 2022 Feb 3;17(2):e0263478. doi: 10.1371/journal.pone.0263478 (PMC8812940; doi:10.1371/journal.pone.0263478)
Supplement: S1 Table — B, unstandardized partial regression coefficient; DNAmPAI-1, DNA methylation-based plasminogen activator inhibitor-1; SE, standard error. Multiple linear regression analysis was performed with PAI-1 as the response variable and phenotype, sex, and age as the explanatory variables. Dummy variables were used as follows: phenotype, control = 0 and autism spectrum disorder = 1; sex, male = 0 and female = 1. Boldface type indicates statistical significance. (DOCX) [file pone.0263478.s005.docx]

**Supplementary Table S1. Multiple-linear regression analysis of DNAmPAI-1.**

|  | Explanatory variable | |  |  |  |  |  |  |  |  |  |
| --- | --- | --- | --- | --- | --- | --- | --- | --- | --- | --- | --- |
|  | Phenotype |  |  |  | Sex |  |  |  | Age |  |  |
| Response variable | B | SE | *P*-value |  | B | SE | *P*-value |  | B | SE | *P*-value |
| DNAmPAI-1 |  |  |  |  |  |  |  |  |  |  |  |
| 1st cohort | 0.967 | 0.364 | **0.0100** |  | −0.089 | 0.362 | 0.807 |  | 0.089 | 0.028 | **0.00207** |
| 2nd cohort | 0.678 | 1.219 | 0.588 |  | NA |  |  |  | 0.154 | 0.068 | **0.0413** |

B, unstandardized partial regression coefficient; DNAmPAI-1, DNA methylation-based plasminogen activator inhibitor-1; SE, standard error.

Multiple linear regression analysis was performed with PAI-1 as the response variable and phenotype, sex, and age as the explanatory variables. Dummy variables were used as follows: phenotype, control = 0 and autism spectrum disorder = 1; sex, male = 0 and female = 1. Boldface type indicates statistical significance.
