# Supplementary material for: Epigenetic clock analysis and increased plasminogen activator inhibitor-1 in high-functioning autism spectrum disorder
Source: PLoS One. 2022 Feb 3;17(2):e0263478. doi: 10.1371/journal.pone.0263478 (PMC8812940; doi:10.1371/journal.pone.0263478)
Supplement: Attachment — Submitted filename: Response to Reviewers.docx [file pone.0263478.s006.docx]

We greatly appreciate the review of our paper entitled “Epigenetic clock analysis and increased plasminogen activator inhibitor-1 in high-functioning autism spectrum disorder”: Manuscript ID PONE-D-21-33732_R1”. The comments from the reviewer were helpful to improve and polish our paper. We have revised our paper according to the comments and marked added texts made in the revised manuscript in red for better readability. Point by point replies are below for the reviewers.

**Reviewer #1:**

In this manuscript, the authors investigated epigenetic age acceleration associated with autism spectrum disorder (ASD) using two independent datasets from blood samples consisting of adult patients with high-functioning ASD and controls. As results, they could not find significant difference in epigenetic age acceleration between ASD and controls in both cohorts. On the other hand, they found a significant increase of DNA methylation-based plasminogen activator inhibitor-1 (DNAmPAI-1) level in individuals with ASD compared to controls in the 1st cohort but not in the 2nd cohort. Finally, they performed a meta-analysis of DNAmPAI-1, and confirmed the significant increase of DNAmPAI-1 in ASD. Furthermore, they discussed the association PAI-1 and ASD.

The content of this manuscript was not suitable for the title and objective.

If they focus the association PAI-1 and ASD, they should change the title and objective of this manuscript.

**Response**: Thank you for your valuable comment. We revised the title as follows:

- Epigenetic clock analysis and increased plasminogen activator inhibitor-1 in high-functioning autism spectrum disorder

(Page 1)

If they focus epigenetic age acceleration associated with ASD, they should perform meta-analysis of epigenetic clock analysis and discuss the differences between the previous report which found the epigenetic age acceleration of ASD (McEwen LM, et al., 2020) and their study.

**Response**: Thank you for your important pointing. As you indicated, McEwen LM, et al previously reported that epigenetic age is accelerated in children with ASD. Our results appear to be inconsistent with theirs. They used the Pediatric-Buccal-Epigenetic clock based on buccal epithelium cells. In contrast, we used other epigenetic clocks based on blood samples. The differences between our and their findings may be due to difference in the type of tissues used for the development of each epigenetic clock. We revised the Discussion as follows:

- McEwen LM, et al previously reported that epigenetic age is accelerated in children with ASD [13], which is inconsistent with our present findings. They used the Pediatric-Buccal-Epigenetic clock based on buccal epithelium cells. In contrast, we used other epigenetic clocks based on blood samples. The differences between our and their findings may be due to difference in the type of tissues used for the development of each epigenetic clock.

(Page 14)

- Finally, only blood samples were analyzed. Future studies using other tissue and cell types, such as neuronal/glial cells and buccal epithelium cells, are required.

(Page 16)

In addition, they should consider other factors affected the alteration of DNA methylation such as medical condition (ie. metabolic problems), prescription, real smoking condition and so on.

**Response**: Thank you for your valuable comment. We additionally performed correlation analysis between epigenetic age accelerations/PAI-1 levels and IQ, ADOS, and ASSQ-R scores in individuals with ASD. We found that GrimAge acceleration was significantly positively correlated with ADOS total in both the 1st cohort (*rho* = 0.415, *p* = 0.0131) and the 2nd cohort (*rho* = 0.886, *p* = 0.0333), but did not show the Bonferroni correction level (corrected significance was defined as *p*-value < 0.05/5 = 0.01) in either cohort. We have revised the manuscript as follows:

- The relationship between continuous variables was analyzed with Spearman’s rank correlation coefficient.

(Page 9)

- We additionally performed correlation analysis between epigenetic age accelerations/PAI-1 levels and IQ, ADOS, and ASSQ-R scores in individuals with ASD. We found that GrimAge acceleration was significantly positively correlated with ADOS total in both the 1st cohort (*rho* = 0.415, *p* = 0.0131) and the 2nd cohort (*rho* = 0.886, *p* = 0.0333), but did not show the Bonferroni correction level (corrected significance was defined as *p*-value < 0.05/5 = 0.01) in either cohort (S4 Fig).

(Page 13)

- We additionally observed a modest significant correlation between GrimAge acceleration and ADOS total in both the 1st and 2nd cohorts; however, these results did not show Bonferroni correction level. Therefore, it was not possible to draw a definitive conclusion. Further studies with a larger sample size are required.

(Page 14)

- **S4 Fig. Relationship between epigenetic clock acceleration/PAI-1 and IQ, ADOS, and ASSQ-R scores in individuals with ASD.**

Scatter plots show epigenetic clock acceleration/PAI-1 vs. IQ, ADOS, and ASSQ-R scores. The relationship was analyzed with Spearman’s rank correlation coefficient.

ADOS, the Autism Diagnostic Observation Schedule; ASD, autism spectrum disorder; ASSQ-R, the high-functioning Autism Spectrum Screening Questionnaire; CTL, control; DNAmTL, DNA methylation-based telomere length; IQ, intelligence quotient; PAI-1, plasminogen activator inhibitor-1.

(Page 21)

**Response**: Regarding other medical conditions, our samples have no real smoking history and no use psychotropic medication. We revised the Methods as follows. Unfortunately, we don't have any information about other diseases or metabolic problems including body mass index. We added the limitation in the Discussion as follows.

- We excluded participants with an IQ < 80, additional psychiatric/neurologic disorders, other medical disorders, history of cigarette smoking, or use of psychotropic medication for at least three months before the blood collection in both ASD and control groups.

(Page 7)

- Fourth, information about other diseases or metabolic problems such as body mass index was lacking.

(Page 16)

**Reviewer #2:**

The authors investigated whether biological aging is observed in adult high-functioning ASD individuals based on age prediction from genome-wide DNA methylation profiles using DNAs extracted from whole blood. The participants in the two independent cohorts (1st: 38 ASD cases vs. 31 controls; 2nd: 6 vs. 10) were Japanese, non-smokers, recruited from a single university hospital, had no comorbidity, no psychotropic medication in at least three months prior to blood collection, and were phenotyped appropriately on the standardized assessment tools. The controls were recruited from the same site and were matched for age, sex, and IQ. The methylation analysis were also performed using the well-documented method, and the statistics were processed appropriately. As a result, they observed a non-significant trend in GrimAge acceleration and a significant difference in DNAmPAI-1 levels between ASD and control groups in the 1st cohort but not in the 2nd cohort. The meta-analysis showed significantly increased DNAmPAI-1 levels in individuals with ASD compared to controls. Although this study has some limitations such as small sample size and lack of plasma PAI-1 levels, to the best of my knowledge, it is the first report of epigenetic clock analysis of whole blood DNA in ASD.

1, P3 L12 "HorvahAge" -> "HorvathAge"

**Response**: Thank you for your comment. We revised the Abstract as follows:

- ... HorvathAge, ...

(Page 2)

2, P16 L5 The fact that there are no racial differences in the samples is also a strength, so it would be good to describe it in the method.

**Response**: Thank you for your valuable comment. We revised the Methods as follows:

- All participants were of Japanese descent and there are no racial differences in the samples.

(Page 7)

**Journal Requirements:**

2. You indicated that you had ethical approval for your study. In your Methods section, please ensure you have also stated whether you obtained consent from parents or　guardians of the minors included in the study or whether the research ethics committee or IRB specifically waived the need for their consent.

**Response**: Our cohorts consist of adult patients with high-functioning ASD and controls. We revised the Methods as follows:

- Briefly, we used two independent cohorts consisting of adult patients with high-functioning ASD and controls; the 1st cohort comprised 38 ASD cases and 31 controls, and the 2nd cohort was six ASD cases and 10 controls.

(Page 7)
